# Genetic and Structural Analyses of Coronaviruses: Insights into SARS-CoV-2 and Beyond

**DOI:** 10.3390/biom16020295

**Published:** 2026-02-13

**Authors:** Miriana Quaranta, Stefano Pascarella

**Affiliations:** Dipartimento di Scienze Biochimiche “A Rossi Fanelli”, Sapienza Università di Roma, 00185 Rome, Italy; miriana.quaranta@uniroma1.it

Coronaviruses are a family of positive-sense RNA viruses, many of which act as etiological agents of human diseases ranging in severity from the common cold to severe pneumonia. The most prominent member is SARS-CoV-2 [1], which triggered a global pandemic in 2020 and profoundly disrupted human activity for several years. While the exact origins of the COVID-19 outbreak remain a subject of ongoing investigation, it is widely attributed to a zoonotic spillover event, likely involving species such as bats or pangolins [2].

The immediate threat posed by the high contagiousness and pathogenicity of SARS-CoV-2 prompted an unprecedented global scientific mobilization. This effort aimed to rapidly decode the virus’s molecular and pathogenic characteristics to inform containment strategies and urgent therapeutic interventions. The resulting volume of scientific literature is staggering: a PubMed search for “SARS-CoV-2 or COVID-19” yields approximately 500,000 publications since 2020—nearly rivaling the total number of papers on “HIV or AIDS” (approximately 550,000) published since 1990. Even more impressive is the number of structural studies on the SARS-CoV-2 proteins: a simple query of the Protein Data Bank using the terms “SARS-CoV-2 or COVID-19” retrieves about 2300 files. Once more, in the same data bank, the query “HIV or AIDS” finds 91 files. Cryo-electron microscopy is the technique that gave the most significant contribution to the huge and fast accumulation of structural data on SARS-CoV-2 proteins and complexes [3].

Research has dissected the virus properties from diverse perspectives, including evolution, molecular biology, genetics, and epidemiology, among others [4,5]. This extensive body of knowledge has guided the search for innovative therapeutic strategies, including the development of new drugs and vaccines. Moreover, the rapid evolution of the SARS-CoV-2 genome and the continuous emergence of new variants provide a theoretical framework for analyzing the dynamics of protein evolution in general and in relation to their functional modulation [5]. All these studies have relied on a vast range of different techniques, from the classical experimental cellular, virological, and epidemiological methods to the most advanced theoretical approaches [6,7,8]. This wealth of knowledge not only clarifies most of the SARS-CoV-2 biology but also provides a generalized template for interpreting other viral pathologies and implementing strategies for prediction and containment of expected future pandemics [9]. In addition, the emergence of COVID-19 pushed a rapid technological advancement of therapeutic methods still under development [10]. A landmark example is the clinical implementation of mRNA vaccine technology, which is now being explored for broader applications, including oncology [11]. Despite all these advancements, significant gaps in our understanding of SARS-CoV-2 remain.

This Special Issue offers a comprehensive overview of the virus’s broad scientific impact. At the same time, it showcases the diverse methodologies, experimental or theoretical, and analytical approaches employed to decipher SARS-CoV-2 biology. Molecular-level investigations are central to this collection, as they unravel the fundamental mechanisms of life and lay the groundwork for designing targeted, effective therapies. Across various perspectives, these nine contributions explore the molecular underpinnings of the virus; while the majority focus on the spike protein and its implications, the final study addresses the frequently overlooked E protein. In addition, these studies provide a perspective on the many open questions that still need to be explored further and indicate directions for future research.

## Overview of the Contributions

Lefebvre et al. (article 1) provide a compelling review of the intramolecular signaling mechanisms within the SARS-CoV-2 spike protein. The authors characterize the interaction between the ganglioside-binding domain (GBD) of the N-terminal domain (NTD) and lipid rafts, detailing how the resulting conformational changes trigger the unmasking of the Receptor Binding Domain (RBD) in the adjacent subunit. Notably, they observe that despite the mutational variability of the NTD interface across successive variants, the interaction is preserved through adaptations in surface electrostatic potential and the formation of specific hydrogen bonds. A conserved residue triad (Q134/F135/N137) was identified on the NTD surface, connected to the RBD contact point of the neighboring protomer via a structural continuum of amino acid residues. This pathway likely facilitates the propagation of allosteric signals upon raft binding. Owing to its conservation across variants, this triad represents a promising target for antiviral interventions aimed at disrupting the intramolecular signaling necessary for viral entry. This study exemplifies how viral evolution can yield profound insights into structure–function relationships, particularly regarding the complexities of allosteric intramolecular communication in protein modulation.

Yamamoto and Noguchi (article 2) review the structural basis of antibody resistance across several SARS-CoV-2 Omicron variants. The authors synthesize research detailing how mutations within the Receptor Binding Domain (RBD) and S2 domain impair interactions with specific antibodies. Notably, they emphasize that the less frequent S2 mutations—specifically in the HR1/HR2 regions—can indirectly modulate the sensitivity of the RBD and NTD to antibody binding by altering spike protein stability and dynamics. This provides another compelling example of intramolecular signaling and highlights the complexity of the spike protein’s molecular properties. Finally, the authors discuss how these structural insights can guide the optimization of monoclonal and bispecific antibodies to overcome resistance in emerging variants.

Mandalari et al. (article 3) report a study aimed at elucidating the interaction between a model compound, the heparan sulfate hexasaccharide (hexa), and the RBD of the WT and Omicron SARS-CoV-2 variants. The model compound mimics the cell surface heparan sulfate (HS), which is organized in proteoglycans or glycoproteins, expressed ubiquitously on the cell surface and within the extracellular matrix. Experimental evidence suggests that the SARS-CoV-2 spike utilizes HS as an auxiliary attachment factor. In this study, the authors apply a combination of molecular dynamics simulations and NMR experiments to identify the site and the modes of hexa binding on the RBD. This approach enables them to explain the molecular basis of the increased affinity of HS in Omicron with respect to wild-type SARS-CoV-2. Moreover, they suggest that the glycosylation of the highly conserved residue N343 can interfere with HS interactions. Overall, this work highlights the value of theoretical approaches such as molecular dynamics in complementing classical experimental methods for the characterization of molecular systems.

Zvartau-Hind et al. (article 4) investigate how hypoxia exacerbates the inflammatory response during coronavirus infection using transcriptomic and proteomic analyses. By employing HCoV-OC43 as a surrogate for SARS-CoV-2, the authors explored the molecular mechanisms underlying the ‘cytokine storm’ characteristic of severe COVID-19. Their analysis of differential gene expressions under hypoxic versus normoxic conditions revealed that infection triggers a significant enrichment of inflammatory pathways. Crucially, this effect is markedly more pronounced under hypoxia. These findings suggest that hypoxia acts as an amplifier of the cellular response to viral infection. Specifically, the study identifies VEGF and CCL20 as key therapeutic targets, as both are synergistically upregulated by the combination of hypoxia and infection. Consequently, modulating hypoxia-signaling pathways—such as the HIF-1 pathway—could provide a viable strategy to mitigate hyperinflammation and prevent cytokine storms in severe cases.

Fuochi et al. (article 5) report on an investigation into the potential use of heparan sulfate (HS) and enoxaparin (EX)—a low-molecular-weight heparin—as inhibitors of viral infection. These molecules represent promising therapeutic candidates because their mechanisms of action are independent of the specific mutational landscape of the spike protein. The study demonstrates that HS and EX effectively inhibit SARS-CoV-2 pseudoviral entry through distinct mechanisms: HS acts as a competitive decoy at the host cell surface—consistent with the findings in article 3—by mimicking endogenous glycosaminoglycans and sterically hindering spike interactions. In contrast, EX interferes with ACE2 interaction through direct binding to the viral spike protein. These findings support the hypothesis that HS and EX could be utilized, potentially at low doses, as effective agents for intranasal or topical administration in preventive or early therapeutic contexts. Furthermore, structural models suggest that the efficacy of HS and EX is likely to be preserved against emerging variants. Overall, studies of this nature have broad significance, as they pave the way for innovative strategies to prevent infections from other respiratory viruses.

Gheeraert et al. (article 6) present a comprehensive theoretical investigation into the interaction between the human ACE2 receptor and the spike proteins of six SARS-CoV-2 variants: WT, Delta, Omicron BA.1, BA.1 Q493K, BA.2, and BA.4/BA.5. Utilizing long-timescale molecular dynamics (MD) simulations of ACE2-RBD complexes, the authors performed a detailed analysis of trajectories and intermolecular interactions. Their study identifies specific structural patches on the ACE2 and RBD surfaces involved in electrostatic and hydrophobic interactions, delineating the role of key mutations. The findings suggest that Omicron variants exhibit a stronger affinity for ACE2 compared to the Delta variant and the original strain. This work provides new insights into the dynamic binding of the spike RBD, potentially explaining the competitive advantage of Omicron. Furthermore, the authors propose that coronaviruses possess a vast “conformational reservoir,” allowing them to maintain human ACE2 affinity while evolving to escape antibody pressure. Ultimately, this study reaffirms that MD simulations—despite their intrinsic limitations—can uncover structural phenomena not evident in static models, serving as a vital complement to experimental structural data.

Burkova and Bakhno (article 7) review current knowledge on the intracellular trafficking of the SARS-CoV-2 spike protein, focusing on how its localization is dictated by specific molecular signals within the C-terminal tail. The authors summarize research investigating the role of the cytoplasmic tail (CT) in protein transport and its delivery to the plasma membrane.

The cytoplasmic domain contains structural binding motifs for the proteins COPI, COPII, and SNX27, all of which are essential for the intracellular trafficking of the spike glycoprotein. Furthermore, a binding motif for the ERM protein family is located within the cytoplasmic moiety; the interaction with ERM proteins, which mediate cytoskeletal linking, is crucial for viral infection progression. Additionally, the CT contains cysteine residues that undergo S-palmitoylation, a process necessary for the stable integration of the spike protein into the plasma membrane.

As highlighted by the authors, elucidating these trafficking mechanisms is fundamental for optimizing mRNA or adenovirus vector vaccines that encode the full-length spike protein. During synthesis, a significant portion of the spike protein tends to accumulate in intracellular compartments; however, only surface-exposed antigens are recognized by B-cell receptors to trigger antibody production. Consequently, enhancing antigen expression at the cell surface could enable dose sparing, thereby reducing the risk of adverse effects. This review highlights key studies that advance this objective.

Alshahrani et al. (article 8) provide an analysis of the structural basis underlying the evolution of antibody (Ab) escape capability in Omicron variants, such as JN.1. They employ an extensive theoretical framework, relying primarily on molecular dynamics and molecular modeling, to investigate the interaction between two classes of antibodies, E1 and F3, and their corresponding spike RBD epitopes. The authors examined how evolutionary pressures drive the emergence of variants capable of effectively evading Ab binding.

The results indicate that the binding of E1-class Abs is determined by a limited number of key hotspots: T345, R346, and K444. At these sites, the synergistic contribution of van der Waals forces and electrostatic interactions is crucial for stable binding. In contrast, binding by F3-class antibodies is driven predominantly by van der Waals interactions. Overall, these observations suggest that SARS-CoV-2 variants evolve through mutations that balance antibody escape with maintained ACE2 affinity. This dual evolutionary pressure is central to viral fitness and immune evasion. The outcomes of this theoretical approach are consistent with experimental data and provide a robust framework for their interpretation.

Volovik et al. (article 9) investigate the enigmatic and understudied E viroporin to elucidate how the membrane lipid environment influences its structure and function. The study further examines the role of specific SARS-CoV-2 E protein domains in modulating membrane curvature and ion permeability. By employing fluorescence confocal microscopy, patch-clamp recordings, and atomic force microscopy, the authors demonstrate the protein’s lipid-dependent behavior. Specifically, while the E protein shows negligible binding to uncharged membranes, its interaction with anionic lipid bilayers is sensitive to cholesterol levels: it assembles as a dimer at low cholesterol concentrations, whereas higher concentrations promote the formation of pentamers with distinct functional properties.
